# A novel form of human disease with a protease-sensitive prion protein and heterozygosity methionine/valine at codon 129: Case report

**DOI:** 10.1186/1471-2377-10-99

**Published:** 2010-10-25

**Authors:** Ana B Rodríguez-Martínez, Joseba M Garrido, Juan J Zarranz, Jose M Arteagoitia, Marian M de Pancorbo, Begoña Atarés, Miren J Bilbao, Isidro Ferrer, Ramón A Juste

**Affiliations:** 1 Department of Animal Health, Neiker-Tecnalia, Berreaga 1, 48160 Derio, Bizkaia, Spain; 2 Neurology Service, Hospital de Cruces, Plaza Cruces-gurutzeta 12, 48902 Barakaldo, Bizkaia, Spain; 3 Department of Health and Consumption, Gobierno Vasco, San Sebastian-Donostia Kalea 1, 01010 Vitoria-Gasteiz, Alava, Spain; 4 Department of Zoology and Animal Cellular Biology, Paseo Universidad 7, Universidad del País Vasco, 01006 Vitoria-Gasteiz, Alava, Spain; 5 Pathology Service, Hospital de Txagorritxu, José Achótegui s/n, 01009 Vitoria-Gasteiz, Alava, Spain; 6 Neurology Service, Hospital de Mendaro, Mendarozabal s/n, 20850 Mendaro, Guipúzcoa, Spain; 7 Institut de Neuropatologia, Servei Anatomia Patològica, IDIBELL-Hospital Universitari de Bellvitge, Carrer Feixa Llarga s/n, 08907 Hospitalet de Llobregat, Barcelona, Spain

## Abstract

**Background:**

Sporadic Creutzfeldt-Jakob disease (sCJD) is a rare neurodegenerative disorder in humans included in the group of Transmissible Spongiform Encephalopathies or prion diseases. The vast majority of sCJD cases are molecularly classified according to the abnormal prion protein (PrP^Sc^) conformations along with polymorphism of codon 129 of the PRNP gene. Recently, a novel human disease, termed "protease-sensitive prionopathy", has been described. This disease shows a distinct clinical and neuropathological phenotype and it is associated to an abnormal prion protein more sensitive to protease digestion.

**Case presentation:**

We report the case of a 75-year-old-man who developed a clinical course and presented pathologic lesions compatible with sporadic Creutzfeldt-Jakob disease, and biochemical findings reminiscent of "protease-sensitive prionopathy". Neuropathological examinations revealed spongiform change mainly affecting the cerebral cortex, putamen/globus pallidus and thalamus, accompanied by mild astrocytosis and microgliosis, with slight involvement of the cerebellum. Confluent vacuoles were absent. Diffuse synaptic PrP deposits in these regions were largely removed following proteinase treatment. PrP deposition, as revealed with 3F4 and 1E4 antibodies, was markedly sensitive to pre-treatment with proteinase K. Molecular analysis of PrP^Sc ^showed an abnormal prion protein more sensitive to proteinase K digestion, with a five-band pattern of 28, 24, 21, 19, and 16 kDa, and three aglycosylated isoforms of 19, 16 and 6 kDa. This PrP^Sc ^was estimated to be 80% susceptible to digestion while the pathogenic prion protein associated with classical forms of sporadic Creutzfeldt-Jakob disease were only 2% (type VV2) and 23% (type MM1) susceptible. No mutations in the PRNP gene were found and genotype for codon 129 was heterozygous methionine/valine.

**Conclusions:**

A novel form of human disease with abnormal prion protein sensitive to protease and MV at codon 129 was described. Although clinical signs were compatible with sporadic Creutzfeldt-Jakob disease, the molecular subtype with the abnormal prion protein isoforms showing enhanced protease sensitivity was reminiscent of the "protease-sensitive prionopathy". It remains to be established whether the differences found between the latter and this case are due to the polymorphism at codon 129. Different degrees of proteinase K susceptibility were easily determined with the chemical polymer detection system which could help to detect proteinase-susceptible pathologic prion protein in diseases other than the classical ones.

## Background

Sporadic Creutzfeldt-Jakob disease (sCJD) is a fatal neurodegenerative disorder which constitutes the most common form of human Transmissible Spongiform Encephalopathy (TSE) occurring at a rate of 1-1.5 cases per million of the population per annum[1]. Clinical features may vary but classic sCJD cases present a rapidly progressive dementia accompanied by focal neurological signs that progress to akinetic mutism and death within 4-6 months[2,3]. Neuropathologic hallmarks are neuronal loss, spongiosis and reactive gliosis, which are variable in nature, severity and location[4,5]. Two protease resistant PrP^Sc ^types have been described associated with sCJD, both presenting a three-band pattern: diglycosylated, monoglycosylated and aglycosylated. Type 1 is characterised by an aglycosylated isoform of 21 kDa whereas type 2 isoform is 19 kDa in size[6]. Few sCJD cases have been described with PrP^Sc ^conformations different from type 1 and type 2 after proteinase-K (PK) digestion[7-10]. Two of them were characterised by the absence of the diglycosylated isoform[7,8], while a unique PrP^Sc ^resistant fragment of 6 kDa size was observed in another case [9]. More recently, a novel human disease, defined by the authors as "protease-sensitive prionopathy" (PSPr) has been described. It showed a distinct clinical and neuropathological phenotype and a more sensitive to PK digestion PrP^Sc ^[10]. Patients presented behavioural and psychiatric manifestations and longer duration of the disease and all of them were valine homozygous. Histopathologically, minimal spongiform degeneration with larger vacuoles than in typical sCJD as well as minimal astrogliosis were described. This lesion profile mainly affected the cerebral neocortex, basal ganglia and thalamus. Abnormal PrP was less resistant to PK digestion and it showed a ladder-like pattern on Western blot, with PrP fragments ranging from 29 to 6 kDa, all detected with Mab 1E4.

## Case presentation

### Clinical findings

A 74-year-old man presented to his general practitioner in August 2006 complaining of memory loss and was then referred to the neurologist. He showed a rapid global cognitive decline associated with aggressiveness, bizarre behaviour and language loss. This was accompanied by severe anomia, disinhibition and a score of 10/30 on MMSE. There were no focal signs, myoclonus or ataxia. The clinical deterioration was very rapid and by December 2006 he was in an akinetic-mutism-like syndrome with abnormal posturing. Two cranial magnetic resonance imaging (MRI), in October and December 2006, including T1, T2, FLAIR and DWI sequences, showed moderate signs of brain atrophy but no increase in abnormal cortical or basal ganglia signal. Electroencephalogram (EEG) was non-diagnostic and protein 14-3-3 level in the cerebrospinal fluid (CSF) was normal. The patient died in March 2007. Family history of dementia included an 80-year-old brother diagnosed with probable Alzheimer disease.

### Genetic findings

No mutations were found in the open reading frame after sequencing the prion protein gene (PRNP). A heterozygosis methionine valine (MV) was observed in codon 129.

### Neuropathology

**Figure 1:**
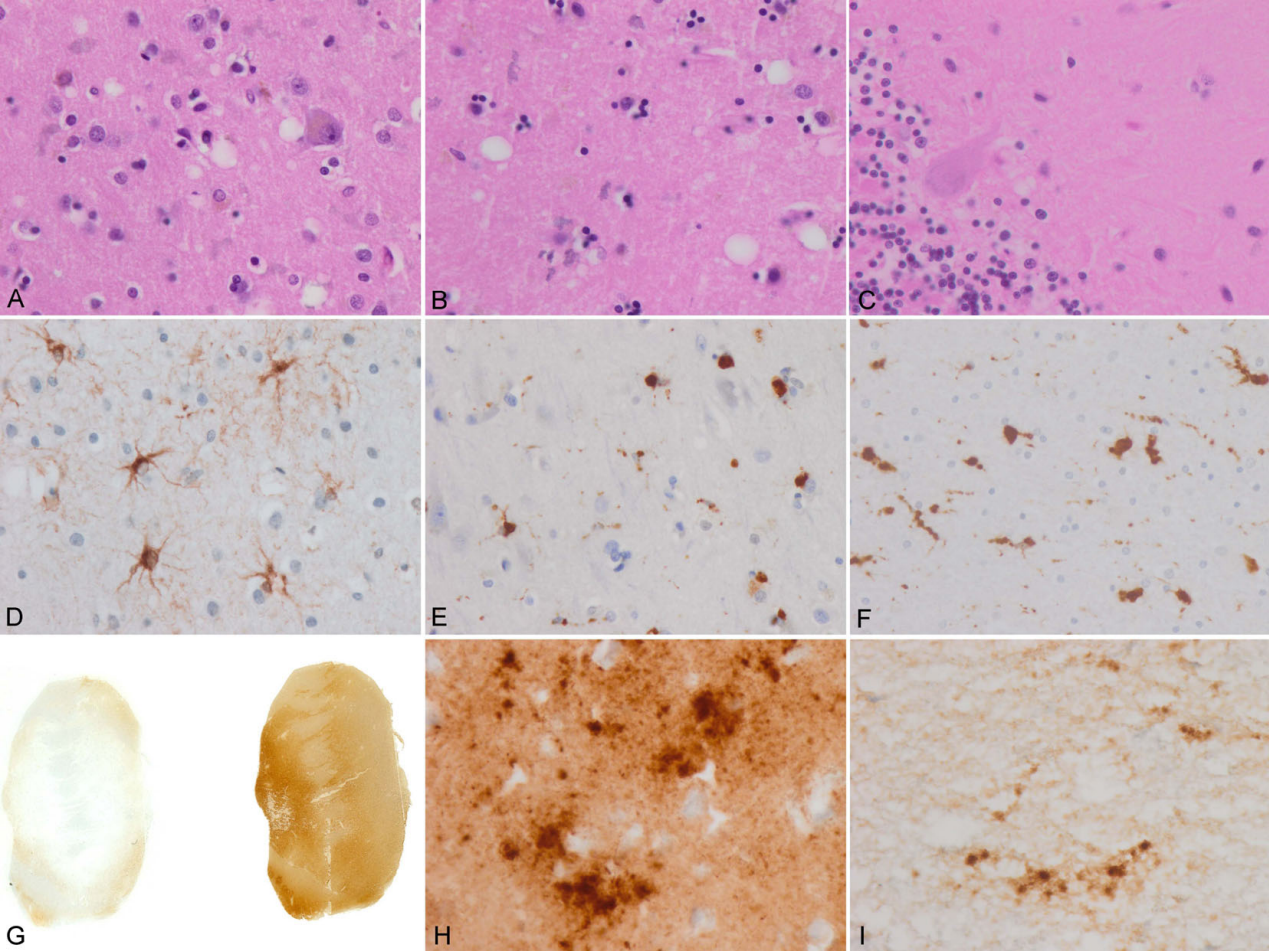
**Main neuropathological findings**. A: Mild spongiform change in the frontal cortex; B: Mild spongiform change in the putamen/globus pallidus characterised by predominance of large vacuoles; C: A few small vacuoles in the vicinity of a Purkinje cell; D: Focal astrocytosis in the cerebral cortex; E and F: Microgliosis with globular reactive microglia in the cerebral cortex and subcortical white matter, respectively; G: PrP immunostaining with and without PK pre-treatment in the putamen/globus pallidus. PrP immunoreactivity practically disappears in PK-treated section. H: PrP immunostaining in the cerebral cortex without PK pre-treatment showing PrP-positive punctate (synaptic-like) deposits and large granular confluent deposits forming coarse plaque-like accumulations; I: A few PrP-immunoreactive granular deposits are seen in sections after PK pre-treatment. A-C: haematoxylin and eosin; D: GAFP immunohistochemistry; E and F: CD68 immunostaining; G-I: PrP immunohistochemistry.

**Figure 2:**
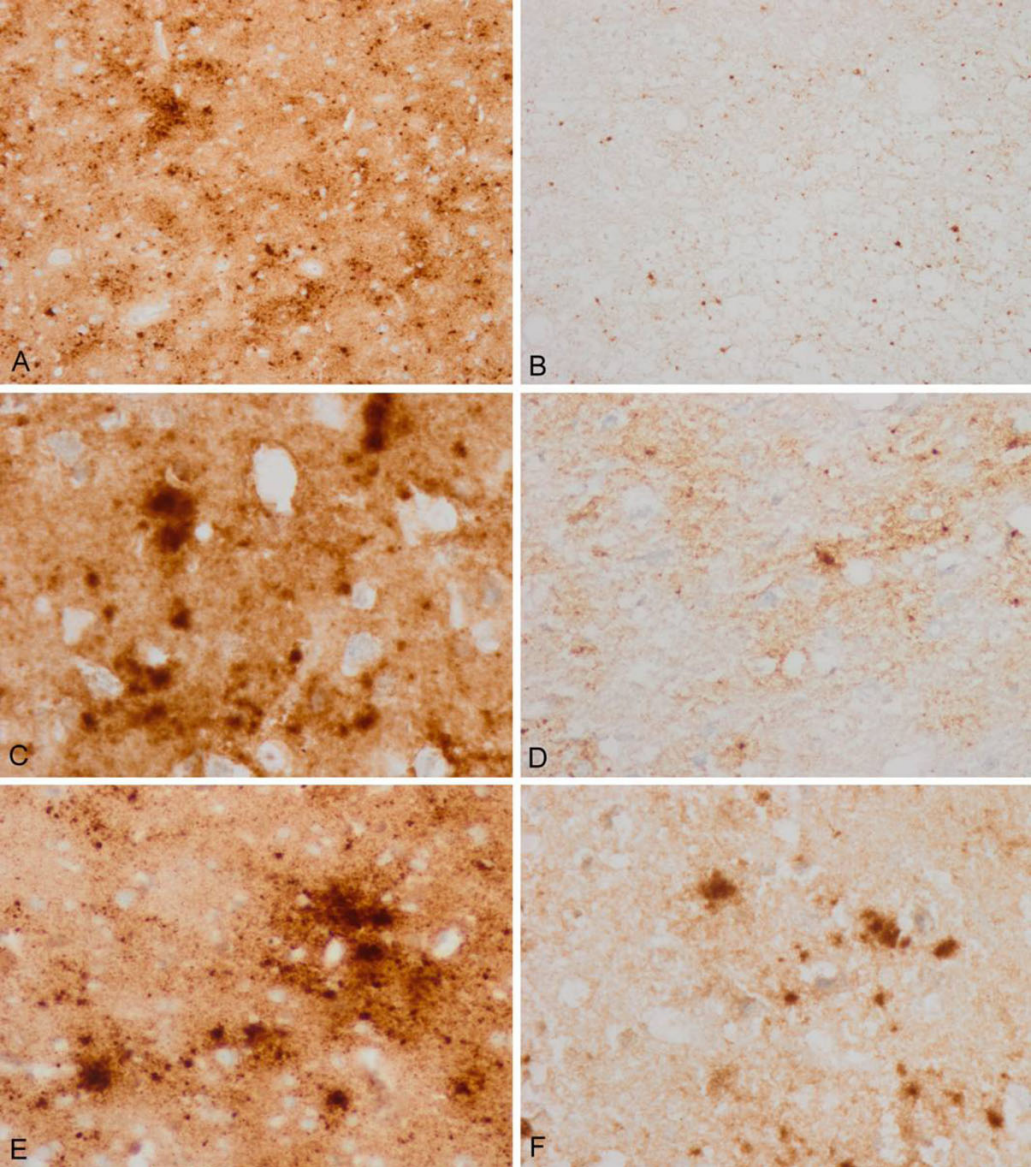
**3F4 immunohistochemistry without and with proteinase K pre-treatment in the same regions of consecutive serial sections**. Parallel (A, B; C, D; and E, F) cortical regions pre-treated with proteinase K (B, D, F) show marked reduction of PrP immunoreactivity when compared with serial sections without proteinase K pre-treatment (A, C, E). Different regions with variable amounts of total PrP were selected in order to have a comprehensive idea of PrP sensitivity.

**Figure 3:**
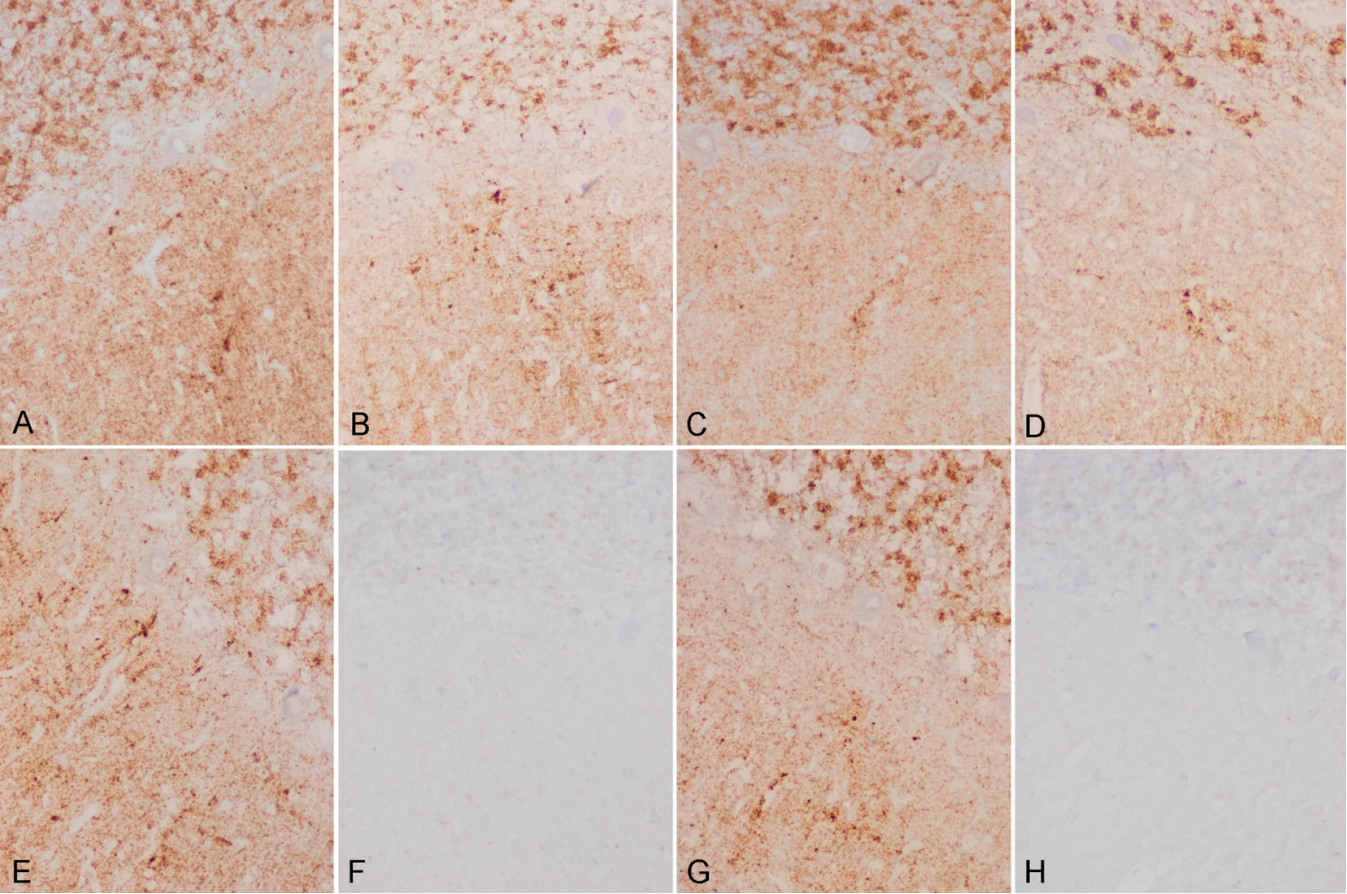
**3F4 and 1E4 immunohistochemistry in MV1 sCJD and the proband**. 3F4 (A, B, E, F) and 1E4 (C, D, G, H) immunohistochemistry without (A, C, E, G) and with (B, D, F, H) PK pre-treatment in the cerebellum of one case of sCJD MV1 with synaptic PrP deposition (A-D) and in the cerebellum of the proband (E-H) show different patterns, when PK-treatment was performed. In the MV1 sCJD case, synaptic PrP deposition, as revealed with 3F4 and 1E4 antibodies, is observed in the molecular and granular layers of the cerebellum. PrP immunoreactivity is largely resistant to the treatment with PK. Synaptic PrP immunoreactivity with the antibodies 3F4 and 1E4 is also found in the molecular and granular layer in the proband; however, immunostaining is lost following incubation with PK. Notice that formic acid treatment did not seem to modify PK susceptibility.

Moderate-to-mild spongiform change was present in the neocortex, putamen/globus pallidus and thalamus, with the lesions being more evident in the putamen and frontal cortex (Figure 1A and 1B). Confluent vacuoles were not found in any region. Except for a few focal vacuoles in the deeper molecular layer, the cerebellar cortex was otherwise unremarkable (Figure 1C). Neurons were largely preserved in the cerebral cortex and basal ganglia although focal astrogliosis was seldom observed (Figure 1D). Mild-to-moderate microgliosis was present in the cerebral cortex and basal ganglia, and subcortical white matter, respectively (Figure 1E and 1F). Immunostaining of PrP without proteinase K pre-treatment showed strong staining characterised by fine punctate deposits (synaptic-like) and irregular granular, often confluent, deposits that could be categorised as diffuse synaptic (Figure 1G and 1H). Perineuronal and cerebellar plaque-like deposits, kuru plaques and florid plaques were absent. Following PK treatment, the vast majority of staining disappeared, except a few granular PrP PK-resistant deposits (Figure 1G and 1I). The cerebellum showed a discrete PrP synaptic-like pattern in the molecular and granular layers which vanished after PK pre-treatment. Sensitivity to PK pre-treatment was best visualized in consecutive sections with and without pre-treatment with PK (Figure 2). Parallel sections stained with the 3F4 antibody showed marked reduction of PrP immunoreactivity, as evaluated by densitometry, involving 70-80% of the total PrP in tissue sections. This was further confirmed by incubating tissue sections with the 1E4 antibody, and comparing the PrP immunohistochemical pattern of one sCJD MV1 case with the proband. As shown in Figure 3, 3F4 and 1E4 synaptic PrP immunoreactivity in the common MV1 case showed resistant PrP immunoreactivity (Figure 3A-D). In contrast, 3F4 and 1E4 immunoreactivity was practically abolished after PK pre-treatment in the proband (Figure 3E-H).

In addition to these changes, neurofibrillary tangles and pre-tangles, as well as granules (grains), were present in the entorhinal and perirhinal cortices, subiculum and CA1 and CA3 regions of the hippocampus. A few pre-tangles and grains were also seen in the amygdala. These changes were accompanied by a few hyper-phosphorylated tau deposits in neurons of the dentate gyrus, coiled bodies in the white matter of the temporal lobe, and peri-ventricular astrocytes. Scattered αB-crystallin-immunoreactive ballooned neurons were present in the entorhinal cortex and amygdala. Tau pathology was consistent with Alzheimer disease stage III and argyrophilic grain disease stage 3. Amyloid plaques and α-synuclein inclusions were absent. No abnormalities were found with anti-TDP-43 antibodies.

### Biochemical analysis

**Figure 4:**
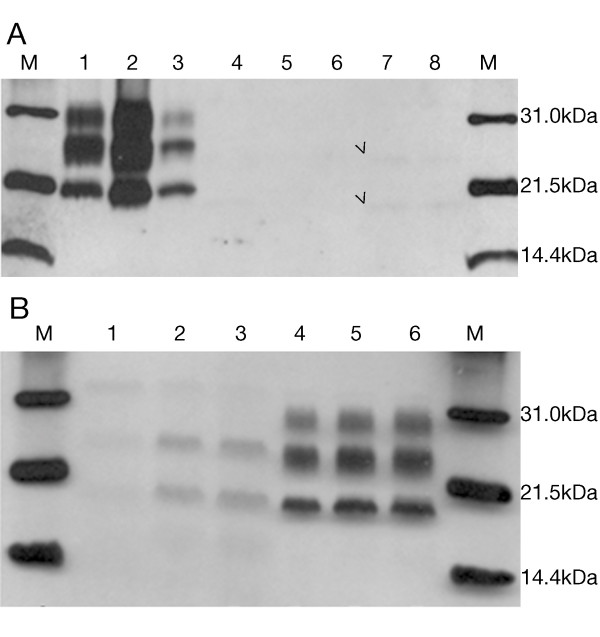
**Immunoblot images under standard conditions**. A. 10% brain homogenate digested with 440 ug/ml PK final concentration and incubated with Mab 3F4. M: Molecular weight marker. 1-3: sCJD MM1 thalamus, frontal and temporal cortex, respectively. 4-8: Occipital cortex, cerebellum, parietal cortex, frontal cortex and temporal cortex of the patient, respectively. Film exposure time: 5 minutes. Arrowheads indicate band position. B. 10% brain homogenate digested with decreasing PK concentration: 440 μg/ml (1, 4), 100 μg/ml (2, 5) and 50 μg/ml (3, 6) and incubated with Mab 3F4. Temporal cortex of the patient (1-3) and occipital cortex of a sCJD VV2 case (4-6).

Standard PrP Western-blot procedure (10% brain homogenate and final PK concentration of 440 μg/ml) failed to detect PrP^Sc^. Increasing the volume loaded into the gel from 5 to 10 μl yielded an extremely weak signal corresponding to 24 and 19 kDa under saturating film exposure times (Figure 4A). Decreasing PK concentrations (440, 100 and 50 μg/ml) showed an increase in the PrP^Sc ^signal, which was suggestive of a PK-sensitive prion protein (Figure 4B). Even then, only two bands of 24 and 19 kDa were visible. After increasing the brain homogenate percentage to 20%, the same two-band pattern was obtained.

**Figure 5:**
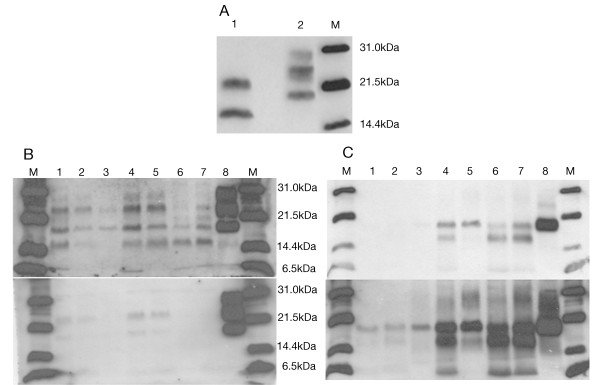
**Immunoblot images under modified methodology**. A. Immunoblot with TeSeE^® ^Western kit of patient occipital cortex (1) and sCJD VV2 control (2) incubated with Mab Sha31. B. Immunoblot of TeSeE^® ^digested and purified samples incubated with Mab 3F4 (upper) and 6H4 (lower). C. PNGase F digestion and detection with Mab 3F4. Film exposure time: 1 minute (upper) and 3 minutes (lower). M: molecular weight marker. 1-8, respectively: occipital cortex, putamen/globus pallidus, cerebellum, parietal cortex, thalamus, frontal cortex, temporal cortex and sCJD VV2 control.

Using the TeSeE^® ^kit, characterised by softer PK digestion conditions followed by steps of purification and concentration of the protein and staining with Sha31 Mab, the presence of two unexpected bands of 21 and 16 kDa was revealed (Figure 5A). This band profile was observed in all the brain regions and it constituted a striking result, since their molecular weight was different from that previously detected with Mab 3F4 and 6H4. Performing a combination of digestion, purification and concentration of the sample according to TeSeE^® ^kit recommendations, along with detection using 3F4 and 6H4, yielded a novel pattern. Not only the previous bands of 24, 21, 19 and 16 kDa were present in each of the samples, but also a very weak band of 28 kDa and a fragment of approximately 6kDa size were observed in some brain regions (Figure 5B). Furthermore, differences of signal intensity were obtained with 3F4 and 6H4 antibodies suggesting differential affinity for PrP^Sc ^which could be interpreted as a different protein conformation in which the 3F4-binding epitope was more exposed than the 6H4 one.

Deglycosylation analysis revealed three aglycosylated isoforms of 19, 16 and 6 kDa, which were more intense in the cortex (parietal, frontal and temporal) and weaker in the occipital cortex and putamen/globus pallidus. In the thalamus region, two bands were detected, a more intense one of 19 kDa and a weaker one of 16 kDa. Finally, the cerebellum was the only region where a single aglycosylated band of 19 kDa was observed (Figure 5C), similar to that found in sCJD type 2. However, we cannot rule out the possibility that this finding was the result of the presence of a small amount of PrP^Sc ^and an underrepresentation of the other bands, as observed in the thalamus, where a 16 kDa size band was only observed under longer film exposure times.

Evaluation of sensitivity to PK digestion was achieved by measuring the absorbance of PrP^Sc ^before and after treatment with proteinase K using the IDEXX HerdChek BSE Test. This technology is based on selective PrP^Sc ^capture by a specific chemical polymer through polyionic interactions in the presence of PrP^C ^from a brain homogenate sample. Absorbance values decrease with serial dilutions, so it can be assumed that the quantity of PrP^Sc ^is directly proportional to the absorbance. The goal of this protocol was to perform relative quantification of PrP^Sc ^without treatment with proteases. We consider that the introduction of a digestion step could be useful to easily evaluate the relative resistance to PK digestion. The results showed that the absorbance values decreased after PK treatment in all the samples (Table 1). For multi-infarct encephalopathy (MIE) and sCJD VV2, the signal detection was reduced in a 4.32% and 2.02%, respectively, but the reductions were not statistically significant. By contrast, samples from the proband and sCJD MM1 showed a statistically significant (p < 0.005) reduction of the signal in a 79.82% and 22.68%, respectively. Absorbance values for MIE were below the cut-off, at the same level as negative controls. The remaining values were above the cut-off.

**Table 1:** Results of the evaluation of proteinase K digestion susceptibility.

Case	PK treatment	Mean	Std. deviation	Signal reduction (%)	P value
Proband	No	1.189	0.698	**79.82**	**<0.0001**
	Yes	0.240	0.140		
					
sCJD MM1	No	3.737	0.206	**22.68**	**0.0041**
	Yes	2.890	1.076		
					
MIE	No	0.062	0.009	4.37	0.2740
	Yes	0.059	0.004		
					
sCJD VV2	No	3.629	0.115	2.02	0.1133
	Yes	3.556	0.152		

Proband: The case reported here; sCJD MM1: Sporadic Creutzfeldt-Jakob disease type 1 methionine homozygous at codon 129; sCJD VV2: Sporadic Creutzfeldt-Jakob disease type 2 valine homozygous at codon 129; MIE: A case of multi-infarct encephalopathy used as a negative control

These differentiated levels of signal reduction are indicative of three levels of resistance to PK digestion: high, intermediate and low. A high resistance to PK digestion would be represented by a low percentage of signal reduction, as observed for sCJD VV2. In this case, the reduction of 2% in the signal would indicate that PK digestion would only degrade a minimal fraction of PrP^Sc^, thus suggesting high resistance of the abnormal prion protein. Intermediate resistance would be represented by a slightly higher percentage of signal reduction as observed in sCJD MM1, in which 22% would point to a higher degradable fraction of PrP^Sc ^than that observed in the previous case. This would represent a protein type only slightly sensitive to degradation with proteases, depending on the brain region. Further investigations are being carried out in order to elucidate whether this level of degradation is associated with MM1 protein type or a phenomenon specific to this subject. Finally, low resistance to PK digestion would be represented by a high percentage of signal reduction, for example the 79% observed in the proband, indicating that a high fraction of PrP^Sc ^is degradable. This suggests the existence of abnormal prion protein types extremely susceptible to protease digestion that might potentially be overlooked by detection methods based on the characteristic proteinase resistance of the pathologic prion protein.

## Discussion

The present report describes a case of a novel human disease with abnormal prion protein sensitive to protease and MV heterozygosity at polymorphic codon 129 of the PRNP gene. A clinical picture of memory impairment as a first symptom, followed by a rapid evolution leading to an akinetic mutism in a 7-month course, was compatible with sCJD [11]. However, post-mortem examinations showed that neuropathological and biochemical findings did not neatly conform to any of the principal subtypes (MM/MV1, VV2, MV2K with kuru type amyloid plaques; MM/MV2C with predominant cortical pathology with confluent vacuoles and perivacuolar PrP staining; MM2T with prominent thalamic pathology and atrophy; and VV1) [2,6,12].

In reference to biochemical and molecular findings, two main striking biochemical features were observed: sensitivity to protease, resulting in an extremely weak PrP^Sc ^signal in immunoblotting, and the multiband profile. Digestion with decreasing concentrations of PK revealed a more PK-sensitive protein than that observed in the control. Further data supporting this was obtained by means of a methodology based on the selective capture of PrP^Sc ^by a specific chemical polymer. We first tested this technique in controls in order to determine whether the anti-PrP specific antibody could detect human PrP^Sc ^strains. After demonstrating that the technique was applicable to human samples, we treated homogenates with PK prior to submitting the samples (PK treated and untreated) to subsequent analyses. Data obtained this way showed that treatment with proteases reduced the absorbance values proportionally to PrP^Sc ^capture. This suggested a degradation of a fraction of PrP^Sc ^molecules, which was minimal when the protein type was highly resistant, intermediate when it was slightly susceptible and variable depending on the brain region, and maximal when it was highly susceptible. Furthermore, treatment with milder PK digestion conditions and detection with Mab 3F4 showed a biochemical profile of five to six bands of 28, 24, 21, 19, 16 and 6 kDa. Differential affinity of antibodies 3F4 and 6H4 for PrP^Sc ^suggested a protein conformation on which epitope-recognising Mab 3F4 was more exposed than that of Mab 6H4. This protein conformation appeared to be different from that of controls since it did not show this unequal affinity for these antibodies. Deglycosylation analysis revealed the presence of up to three aglycosylated isoforms of 19, 16 and 6 kDa, suggesting the coexistence of several PrP^Sc ^strains [13-15].

Recently, a novel human prion disease defined as a protease-sensitive prionopathy (PSPr) was described in 11 cases. It is characterised by a distinct clinical and neuropathological phenotype and by a PrP^Sc ^more sensitive to PK, with a distinctive electrophoretic profile [10]. Our case showed some features compatible with this novel disease [10] such as a family history of dementia, prominent neuropsychiatric symptoms early in the evolution and the absence of specific abnormalities in the ancillary test such as EEG, 14-3-3 protein in CSF and MRI. In contrast, our case had a short clinical course of seven months (versus a median of 20 months in the Gambetti et al. series) [10]. Regarding abnormal prion protein, evaluation of the sensitivity to PK showed that 80% of the detectable abnormal PrP was PK sensitive, with a ladder-like pattern on Western blot as seen in other PSPr cases. However, it differed from them on the earlier age at onset (74 years vs. upper range value 71), the aforementioned clinical course, higher PrP^Sc ^isoforms (three aglycosylated isoforms of 19, 16 and 6 kDa, *versus *two of 20 and 6 kDa) and MV genotype for PRNP 129 polymorphism. Codon 129 appears to be the most reliable factor to explain these dissimilarities, as recently reported in other PSPr MM and MV cases [16,17], and as observed in other human prion diseases [11,18,19]. However, the existence of other unknown factors cannot be discarded.

Regarding the neuropathological findings, the present case differs from common subtypes of sCJD, including those cases presenting with combined molecular subtypes [12,20]. Main involvement of the putamen/globus pallidus, thalamus and cerebral cortex with slight cerebellar involvement, together with a lower band of 19 kDa, is not common in pure MV cases [2,12]. Cases with MV1 show typical synaptic pattern of PrP immunostaining and slight involvement of the cerebellum, whereas MV2 cases exhibit large numbers of kuru plaques [2,12]. Large confluent vacuoles are common in MM2C but these also differ from the moderately large, non-confluent vacuoles observed in the present case. Finally, PrP sensitive to PK, as revealed in immunohistochemical sections, and further validated by molecular studies, does not occur in common subtypes of sCJD [2,12].

Neuropathologically, this form differs from MV2 by its lack of confluent vacuoles. It also differs from VV2 and MV2K in the absence of cerebellar plaque-like deposits and kuru plaques, respectively. Mixed forms MM/MV1+2C and MV2K+2C are also different for analogous reasons. Finally, MM2T, characterised by thalamo-olivary atrophy, and MM2V, characterised by florid plaques, can be clearly distinguished from the present form. The present case bears similarities to MM/MV1 and VV1 although the band pattern of PrP is obviously different. The original report stressed the size of vacuoles as a distinctive feature, with the vacuoles being larger than those currently seen in MM/MV1 and VV1 cases. Small confluent vacuoles were present in the cerebral cortex in the proband. Sparser vacuoles, a bit larger in size, occurred in the putamen/globus pallidus and thalamus, and they differed from the confluent microspongiosis usually seen in MM/MV1 and VV1 cases. However, it is difficult to draw fair conclusions on this point based on our particular case.

In summary, although clinical signs pointed to sCJD, deposition of PrP sensitive to PK digestion and abnormal prion protein with a ladder-like pattern indicated that our case fitted better with a diagnosis of 'protease-sensitive prionopathy'. However, heterozygosis MV in codon 129 of the prion protein gene suggested that it might rather be a novel form of human disease with abnormal prion protein sensitive to protease. From a technical point of view, it should be noted that the use of milder digestion conditions could provide interesting information on the characteristics of 'less frequent' PrP^Sc ^strains involved in human TSEs. Additionally, the application of new methods which allow the detection of PrP^Sc ^without PK digestion could be of great value in evaluating the level of resistance to PK of abnormal prion protein types and the specific relation between relative amounts of PrP^Sc ^and clinical and neuropathological phenotypes.

## Conclusions

A novel form of human disease with abnormal prion protein sensitive to protease was described. Although clinical signs were compatible with sCJD, the molecular subtype with the abnormal prion protein isoforms showing enhanced protease sensitivity and a ladder-like pattern was reminiscent of the 'protease-sensitive prionopathy'. Whether or not the genotypic difference from previously reported PSPr cases influences the clinical and neuropathological phenotype, as well as the prion protein conformation and its profile after digestion with proteinase K, remains elusive. Nevertheless, this case established a significant difference with that form of disease. The introduction of modifications in the analysis and detection methodology, mainly focused on applying milder digestion conditions, is necessary in order to detect these proteinase-sensitive proteins. This could also be complemented by the use of analytical approaches that allow quantification of PrP^Sc ^before and after treatment with PK. In this manner, pathologic prion protein could be further characterised using a new perspective that would help to study the phenotypic variability of human prion diseases.

It should not be overlooked that the method presented herein opens a way to more easily detecting pathologic proteinase-susceptible prions associated with other neurodegenerative diseases.

## Methods

### Clinical findings

The patient was subjected to standard clinical, electroencephalographic and MRI examinations.

### Genetic findings

Analysis of PRNP was performed by standard sequencing methods.

### Neuropathology

Only the brain was removed at autopsy for neuropathological and biochemical examination. Following the recommended safety guidelines, fresh samples from seven areas (occipital, frontal, parietal and temporal cortex, putamen/globus pallidus, thalamus and cerebellum) were processed for neuropathological analyses. Selected samples of the cerebral cortex, putamen/globus pallidus, thalamus, cerebellum and brain stem fixed in 4% formalin were treated with formic acid, and then post-fixed in formalin and embedded in paraffin. De-waxed sections were stained with haematoxylin and eosin and Klüver-Barrera, or processed for immunohistochemistry, following the En Vision+ system method, for glial fibrillary acidic protein (GFAP), CD68 for microglia, hyper-phosphorylated tau epitopes (antibody AT8), 3Rtau and 4Rtau, β-amyloid 1-40 and β-amyloid 1-42, α-synuclein, αB-crystallin, ubiquitin, TDP-43, and prion protein (antibodies 3F4 and 1E4) without and with PK pre-treatment. Densitometry of immunohistochemical sections not counterstained with haematoxylin was analysed by using modified Total Laboratory v2.01 software. Measurements were expressed as arbitrary units in parallel PrP immunostained sections without and with PK pre-treatment. The results were presented as a percentage of decreased immunoreactivity of PK-treated in comparison with PK-untreated sections.

### Biochemical analysis

#### Western blot

Eight brain regions corresponding to cortex (frontal, temporal, occipital and parietal), cerebellum, caudate nucleus, thalamus and putamen/globus pallidus were analysed. 10% and 20% (w/v) brain homogenates were prepared in lysis buffer [21]. The homogenates were cleared by centrifugation at 2100 rpm and 4°C (Heraeus Biofuge Fresco) for 5 minutes. Supernatants were treated with different final concentrations of proteinase K (440 μg/ml, 100 μg/ml and 50 μg/ml) for 60 minutes at 37°C. The reaction was terminated by the addition of Pefabloc SC (Roche-Diagnostics) to a final concentration of 1 mM. An equal volume of 2× loading buffer (modified from [21]: 125 mM Tris-HCL pH 7; 4% SDS; 20% glycerol; 0,02% bromophenol blue; 200 mM DTT) was added and the samples were denatured at 96°C for 8 minutes before electrophoresis on 16% SDS-Tris-glycine gels (5% stacking) for 90 minutes at 150V. The gels were electroblotted onto PVDF membrane (Immobilon-P, Millipore) and blocked as described elsewhere [21]. After a short wash in PBST 1×, membranes were incubated either with anti-PrP monoclonal antibody 3F4 (epitope 109-112: MKHM) (Sigma) (1:20.000) or 6H4 (epitope 144-152: DYEDRYYRE) (Prionics), diluted 1:5.000 for 1 h. Following a washing step in PBST 1× for 45 min, membranes were incubated with an alkaline phosphatase conjugated goat anti-mouse IgG antibody (Sigma) diluted 1:10.000, and secondary against 6H4 (Prionics, dilution 1:5000) respectively in PBST 1× for 1 h at room temperature or at 4°C overnight. After a washing step of 45 min in PBST 1× and equilibration in 200 mM Tris-HCl; 10 mM MgCl2, pH 9,8 [22] for 5 min, membranes were developed in chemiluminescent substrate (CDP-STAR, Tropix) and visualised on X-Omat AR film (Kodak).

In addition, samples were examined by TeSeE^® ^Western Blot (Bio-Rad) following the manufacturer's recommendations. Briefly, 20% brain homogenate was incubated with proteinase K and detergent solution for 10 min at 37°C before addition of buffer B. After a short mixture, samples were centrifuged at 15000 g for 7 min. The pellet was solubilised in 1× loading buffer (see above) by incubating at 100°C for 5 min. Samples were then centrifuged at 15000 g for 15 min and supernatants were denatured at 100°C for 4 minutes before electrophoresis. Electrophoresis separation was performed as described above. Proteins were transferred onto a PVDF membrane at 115V for 60 min and 4°C. Following transfer, the membrane was soaked successively with PBS buffer, ethanol and distilled water, and then saturated for 30 min with blocking solution. The membrane was incubated for 30 min at room temperature with monoclonal antibody Sha31 against epitope YEDRYYRE (145-152, huPrP), diluted 1:10 in PBST. Following a washing step with PBST, the membrane was incubated for 20 min with goat anti-mouse IgG antibody conjugated to horseradish peroxidase diluted 1:10 in PBST. Finally, membranes were developed in chemiluminescent substrate (Western Blotting detection system, ECL, Amersham) and visualised on film.

A combination of both protocols was also used. In such cases, samples were digested and purified according to TeSeE^® ^Western Blot procedure and incubation with monoclonal antibodies 3F4 and 6H4 was performed as described above.

#### Deglycosilation analysis

In order to detect the non-glycosylated isoforms, samples (either proteinase K digested or purified with TeSeE^®^) were subjected to PNGase F (New England Biolabs) digestion overnight at 37°C and PrP^Sc ^was recovered as described elsewhere [23].

#### Evaluation of sensitivity to PK digestion

For the evaluation of sensitivity to proteinase K digestion the IDEXX HerdCheck BSE Test was performed according to the manufacturer's instructions[24] with modifications. Tissue samples from eight brain regions of four cases were analysed. These cases included a sCJD control MM1, sCJD control VV2, MIE as prion disease negative control and the proband. In brief, 0.25 g of tissue was homogenised in a tissue-disruption tube for 2 cycles of 23 seconds at 6500 rpm in a homogenizer. A fraction of brain homogenate was treated with 100 μg/ml proteinase K (Sigma) for 1 hour at 37°C. The reaction was stopped by adding 1 mM Pefabloc SC (Roche). After this, 100 μl of homogenised samples (PK treated and untreated) were then diluted with 25 μl of working plate diluent, mixed by pippeting six times and transferred (100 ul) to the BSE antigen capture enzyme immunoassay plate. The plate was incubated at 34°C for 20 minutes at 200 rpm in a Thermo shaker PHMP-4 (Grant Instruments, Cambridge Ltd) in order to allow the disease-associated conformer (PrP^Sc^) to bind to the immobilised ligand with high affinity. The plate was washed three times with 1X Wash 1 in a Biotek ELx50™Microplate washer to remove unbound materials, including PrP^C^. The plate was then incubated with 100 μl of CC-conjugate for 25 minutes at 34°C and washed five times with 1X Wash 2. Finally, the plate was incubated with 100 μl horseradish peroxidase (HRPO) substrate for 15 minutes at 34°C in the dark prior to reading the optical density at 450 nm and 650 nm with a plate reader (Model SUNRISE, TECAN). Colour development was related to the relative amounts of PrP^Sc ^captured by the ligand immobilised in the microtiter plate well. Negative controls, positive controls and samples were analysed in duplicate. Results were analysed with Magellan V6.3 software (Tecan Austria GmbH). Calculations of negative controls means (NCmean) were automatically made by the software according to the formula (NCmean = (A1 (A_450_-A_650_) +B1 (A_450_-A_650_))/2 where A1 and B1 are the plate wells for negative control. The value corresponding to (A_450_-A_650_) was calculated and applied as a correction factor to the absorbance values of the samples. The statistical significance of mean comparisons was checked with the Student's t-test for independent samples.

## Consent

Written informed consent was obtained from the next of kin of the patient for publication of this case report. A copy of the written consent form is available for review from the editor-in-chief of this journal.

## Competing interests

The authors declare that they have no competing interests.

## Authors' contributions

ABRM carried out the molecular analyses, and drafted the manuscript. ABRM, JMG and RAJ designed the molecular analyses. JJZ helped to draft the neurological and neuropathological sections of the manuscript. JMA provided neurological examination data and helped to draft the manuscript. MMP carried out sequencing analysis. BAP and IFA performed neuropathological studies and IFA drafted the neuropathological section. MJB diagnosed the patient. RAJ coordinated all the information and completed the writing of the final manuscript. All authors read and approved the final manuscript.

## Pre-publication history

The pre-publication history for this paper can be accessed here:

http://www.biomedcentral.com/1471-2377/10/99/prepub
